# A Technology-Based Training Tool for a Health Promotion and Sex Education Program for Justice-Involved Youth: Development and Usability Study

**DOI:** 10.2196/31185

**Published:** 2021-09-30

**Authors:** Nyssa L Snow-Hill, Geri Donenberg, Edward G Feil, David R Smith, Brenikki R Floyd, Craig Leve

**Affiliations:** 1 Center for Dissemination and Implementation Science Department of Medicine University of Illinois at Chicago Chicago, IL United States; 2 Influents Innovations Eugene, OR United States; 3 School of Public Health University of Illinois at Chicago Chicago, IL United States

**Keywords:** health education, sexual behavior, juvenile delinquency, feasibility studies, evidence-based practice, adolescent health services, inservice training, implementation science, organizational innovation, technology, risk reduction behavior, mobile phone, health technology, health promotion, sexual health

## Abstract

**Background:**

Justice-involved youth are especially vulnerable to mental health distress, substance misuse, and risky sexual activity, amplifying the need for evidence-based programs (EBPs). Yet, uptake of EBPs in the justice system is challenging because staff training is costly in time and effort. Hence, justice-involved youth experience increasing health disparities despite the availability of EBPs.

**Objective:**

To counter these challenges, this study develops and pilot-tests a prototype of a technology-based training tool that teaches juvenile justice staff to deliver a uniquely tailored EBP for justice-involved youth—PHAT (Preventing HIV/AIDS Among Teens) Life. PHAT Life is a comprehensive sex education, mental health, and substance use EBP collaboratively designed and tested with guidance from key stakeholders and community members. The training tool addresses implementation barriers that impede uptake and sustainment of EBPs, including staff training and support and implementation costs.

**Methods:**

Staff (n=11) from two juvenile justice settings pilot-tested the technology-based training tool, which included five modules. Participants completed measures of HIV and sexually transmitted infection (STI) knowledge, sex education confidence, and implementation outcomes such as training satisfaction, adoption, implementation, acceptability, appropriateness, and sustainability. PHAT Life trainers assessed fidelity through two activity role plays participants submitted upon completing the training modules.

**Results:**

Participants demonstrated increases in HIV and STI knowledge (t_10_=3.07; *P*=.01), and were very satisfied (mean 4.42, SD 0.36) with the training tool and the PHAT Life curriculum. They believed that the training tool and curriculum could be adopted, implemented, and sustained within their settings as an appropriate and acceptable intervention and training.

**Conclusions:**

Overall, the results from this pilot test demonstrate feasibility and support continuing efforts toward completing the training tool and evaluating it within a fully powered randomized controlled trial. Ultimately, this study will provide a scalable option for disseminating an EBP and offers a more cost-effective and sustainable way to train staff in an EBP.

## Introduction

Over 700,000 children and adolescents were arrested in the United States in 2019 [1], and African American and Latinx populations were disproportionately represented [2]. Justice-involved youth report elevated rates of mental health problems [3-6], substance misuse [7-20], and risky sexual activity [11,21], and compared to non-justice–involved peers, they are more likely to test positive for sexually transmitted infections (STIs) [22]. Incarceration during adolescence is associated with poor long-term outcomes [23-28], yet it is also an ideal opportunity to deliver intervention programs that address frequently co-occurring health problems. Unfortunately, few evidence-based programs (EBPs) exist for justice-involved adolescents, and those that have demonstrated efficacy go largely unused [29,30].

Several factors are implicated in the low rates of adoption, implementation, and sustainment of EBPs [31-35] in youth service settings [33], and these can be understood within the context of the Consolidated Framework for Implementation Research (CFIR) [32]. The CFIR emphasizes five domains that affect implementation and offers direction for addressing barriers and strengthening facilitators. According to the CFIR, EBPs may be seen as too complex to deliver (ie, intervention characteristics) [31], not an agency priority (ie, outer setting) [36], or difficult to integrate into the justice setting and ongoing programming (ie, implementation processes) [37]. The costs and staff time required to train in the EBPs are often viewed as prohibitive, especially in light of high staff turnover as is commonly observed in justice settings (ie, inner setting) [38]. Staff losses require new trainings and additional resources. Efforts to bring EBPs to scale, therefore, merit careful consideration of the EBP’s characteristics, suitability, acceptability, and cost for the justice setting.

One EBP developed for justice-involved youth, PHAT (Preventing HIV/AIDs Among Teens) Life, has demonstrated acceptability, implementation feasibility, and sustained efficacy [21,39]. PHAT Life is an innovative, manualized, and culturally relevant HIV/STI, substance misuse, and mental health EBP [21,39] that was systematically adapted from three Centers for Disease Control and Prevention evidence-based interventions (RHAP [40], Street Smart [41], and Project STYLE [42]) via a series of carefully crafted stages including guidance from key stakeholders, focus groups, pilot tests, youth and adult advisory boards, probation staff and teachers, mental health and substance misuse counselors, and careful attention to context [43]. Guided by a combination of social learning theory [44] and the Social-Personal Framework [45], PHAT Life recognizes the broad ecological context of development and lived experience during adolescence.

PHAT Life is a group-based, 8-session comprehensive health promotion and sex education program delivered over 2 weeks. The curriculum targets broad psychosocial factors implicated in risk behavior, including promoting positive attitudes toward HIV/STI prevention, positive peer norms, self-efficacy to reduce risk, and less substance misuse and sexual risk taking. Activities are designed to improve emotion regulation skills, advanced planning, and safer sex behaviors (eg, consistent condom use). Sessions encourage youth to recognize and take personal responsibility for their health and to identify strategies and behaviors to accomplish short- and long-term goals. PHAT Life was evaluated in a 2-arm group randomized controlled trial and demonstrated positive effects on sexual behavior, aggression, and recidivism [21,39].

Despite these promising effects, PHAT Life has a critical implementation barrier that has impeded dissemination of other EBPs—the cost, time, and effort involved in training (and retraining) facilitators to deliver the program. In its traditional form, PHAT Life training entails a 2-day in-person group-based meeting involving didactic material, role plays, practice, and feedback. A designated expert trainer presents on effective facilitation skills and managing group dynamics, and then systematically reviews each session of the intervention curriculum. The expert provides ongoing technical assistance and annual in-person refresher trainings. These processes present several challenges to wide-scale dissemination. During in-depth interviews with facilitators and administrators who implemented PHAT Life, the curriculum was described positively, but the training model (eg, in-person, group-based) was perceived as unsustainable, particularly in light of high staff turnover (E Rios, unpublished data, 2020).

Web-based technology offers an alternative training model for EBPs that may be more feasible and acceptable, particularly in resource-limited settings like juvenile justice. A technology-based training tool has the potential to address two well-documented implementation barriers. First, it is more cost effective by allowing staff flexibility in when, how, and where they complete the training. Individual staff can engage without assembling in groups, which can mitigate adverse impact on organizational operations. Second, a technology-based training tool allows refreshers and retraining on-demand at no additional cost because the organization can refer staff back to the tool. Understanding whether a technology-based training tool is feasible and acceptable, leads to knowledge of the material and curriculum, engages the learner, and is sufficient to achieve intervention fidelity is critical to move this model forward.

This study features the development and evaluation of a prototype of a technology-based training tool for PHAT Life. We report on learner knowledge and implementation outcomes, including use, adoption, implementation, acceptability, appropriateness, sustainability, satisfaction, and intervention fidelity. We expected trainees to engage with the modules, learn the curriculum and achieve fidelity, and perceive the training tool as satisfactory and feasible for implementing in their youth service settings.

## Methods

### Development of the Technology-Based Training Tool

The technology-based training tool entailed close collaboration between the academic team who created PHAT Life and the industry team who developed the tool. The final prototype uses dynamic multimedia presentations and an interactive self-paced course to teach group facilitator skills and curricular material. Development of the tool followed nine steps. First, the industry team created an outline of the PHAT Life in-person training for translation into the technology-based tool. Using this outline, trained PHAT Life facilitators and research staff produced text to guide video vignettes of intervention activities and demonstrate facilitator skills. A two-person production team traveled to the research site and filmed two expert PHAT Life facilitators delivering the curriculum to 5 mock participants. The videos were then edited to create 25 clips that were subsequently inserted into the training tool as examples of how to deliver the intervention. For the prototype, the team built an introduction and two of the most challenging PHAT Life sessions. This resulted in five distinct modules (see Table 1).

**Table 1:** Training tool modules.

Module title	Module components	Webpages, n
Introduction	PHAT^a^ Life curriculum overview and logistics, interviews with key personnel, adolescent development	6
Being a PHAT Life facilitator	Interview with lead PHAT Life trainer; facilitator interpersonal and professional qualities: maintaining fidelity, being prepared and nonjudgmental, communicating effectively, collaborating with cofacilitators, acknowledging own background and limitations	13
Setup and breakdown	Overview of PHAT Life manual, materials, and activities that occur in all PHAT Life sessions: Body Scan, Outside/Inside Check-In, How Much Do I Want to Be Here, Group & Personal Goals, Action Agreement, Confidentiality, HIV/AIDS Frame, and Parking Lot	27
Session 1: Anatomy and condom basics	Group Road Map, “The Monster” movie, Female and Male Reproductive Anatomy, Condom Types, External Condoms: 10 Step Game, External Condom Practice, and Condom Practice Under the Influence	27
Session 2: Risk and vulnerability	Virus Carrier Handshake; Risk Spectrum; Sexually Transmitted Infections; HIV/AIDS 20 Quick Fire Facts; High Risk, Low Risk, No Risk; and “Walking on Sunshine” movie	26

^a^PHAT: Preventing HIV/AIDS Among Teens.

**Figure 1:**
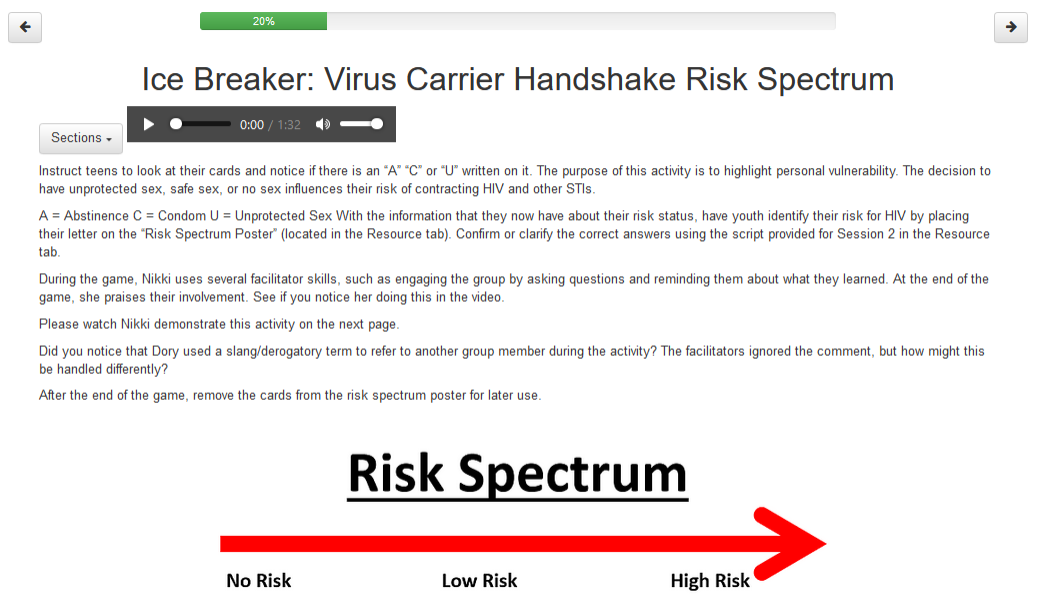
Example of activity on a computer browser with audio narration. STI: sexually transmitted infection.

Next, text was created for each of the five modules based on the PHAT Life curriculum and training materials. The text also described the video clips, pointed out facilitator skills, and brought attention to facilitation strategies. Each web page was reviewed by several team members for ease of access, comprehension, and usability. Once the text was finalized, a team member with voice recording experience created audio narrations to accompany each page and accommodate literacy concerns (see Figure 1).

### Prototype of the Technology-Based Training Tool

**Figure 2:**
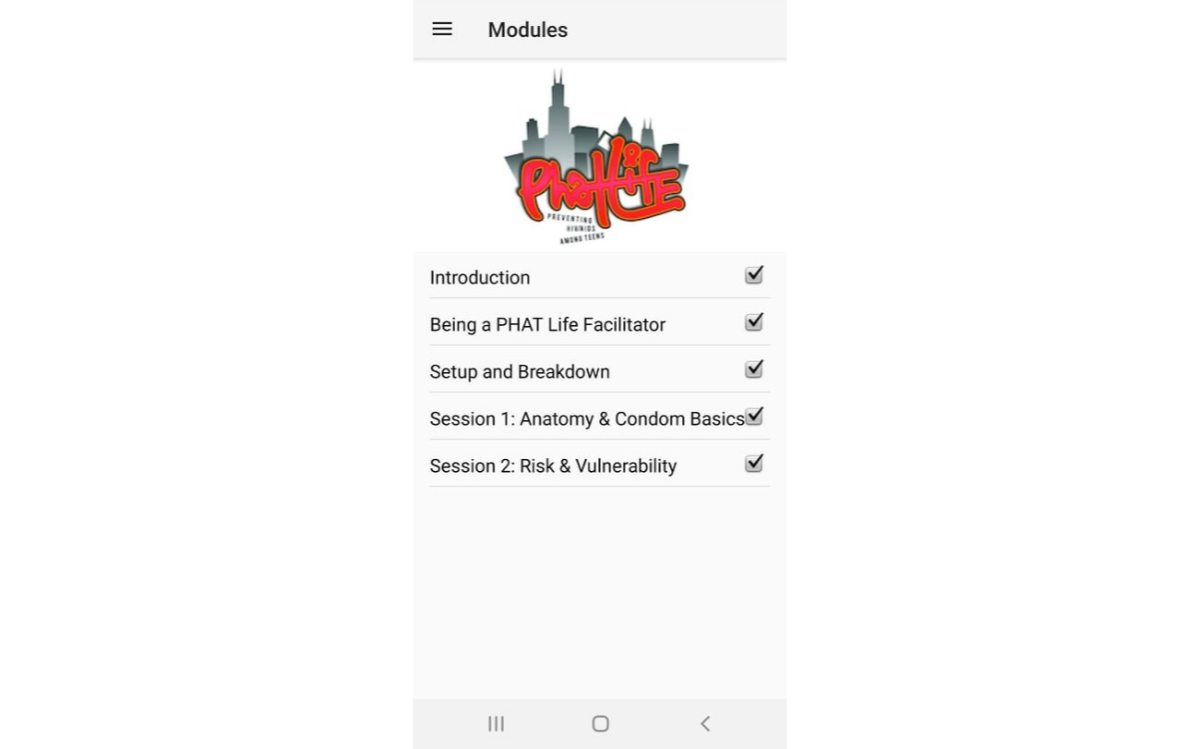
Phone app menu for the five-module training tool. PHAT: Preventing HIV/AIDS Among Teens.

**Figure 3:**
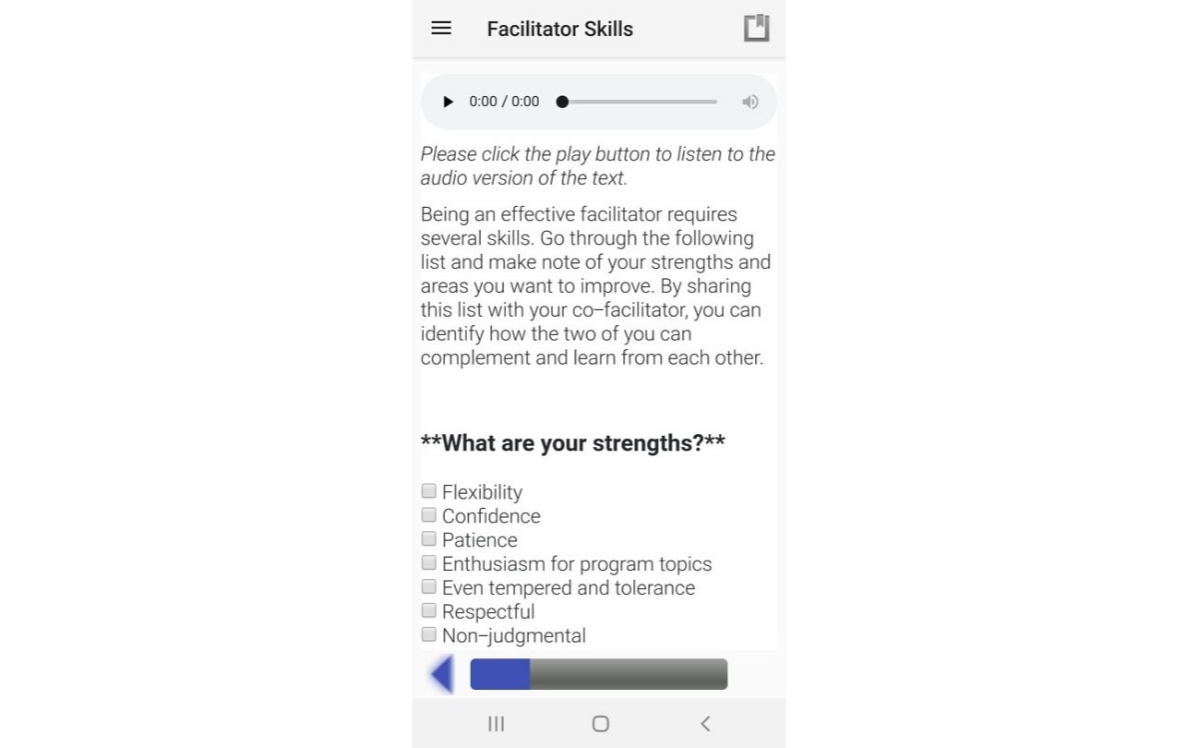
Example of questions embedded within the training modules on the phone app.

**Figure 4:**
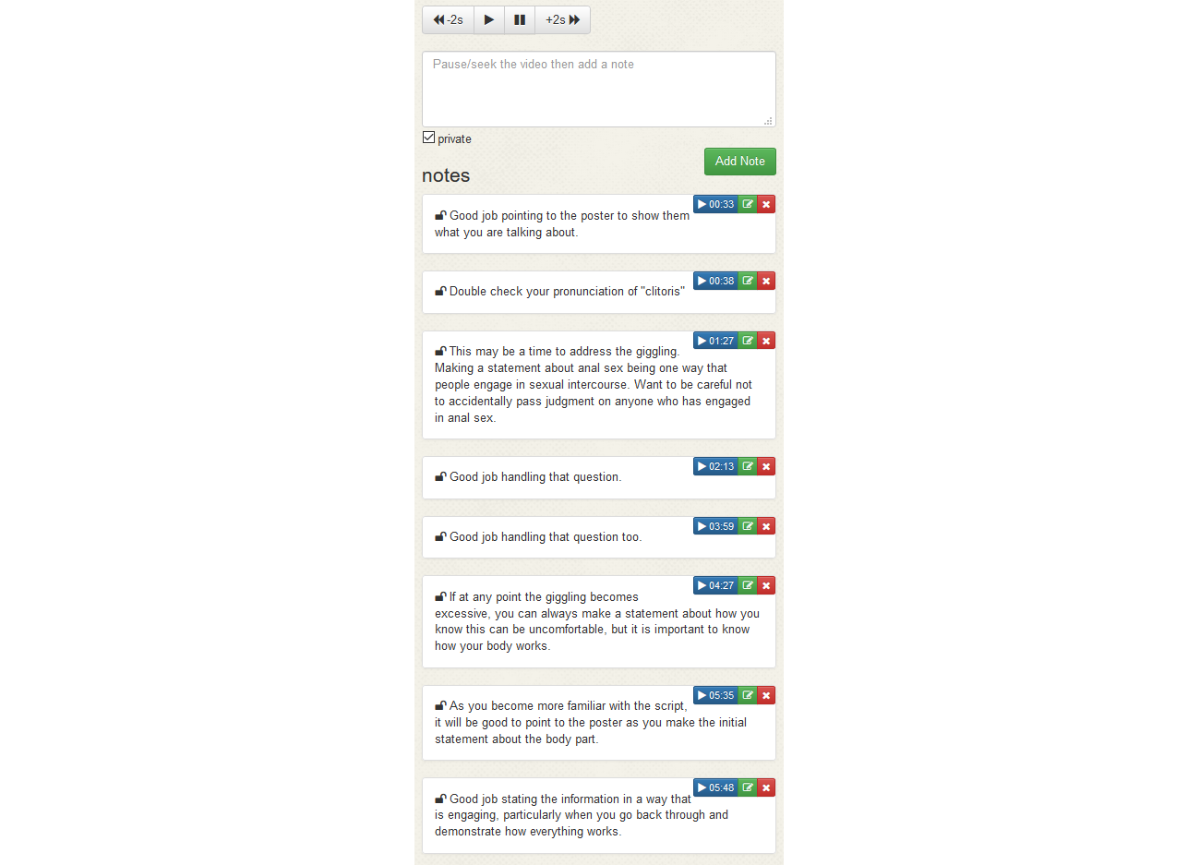
Time-stamped comments connected to uploaded participant videos.

**Figure 5:**
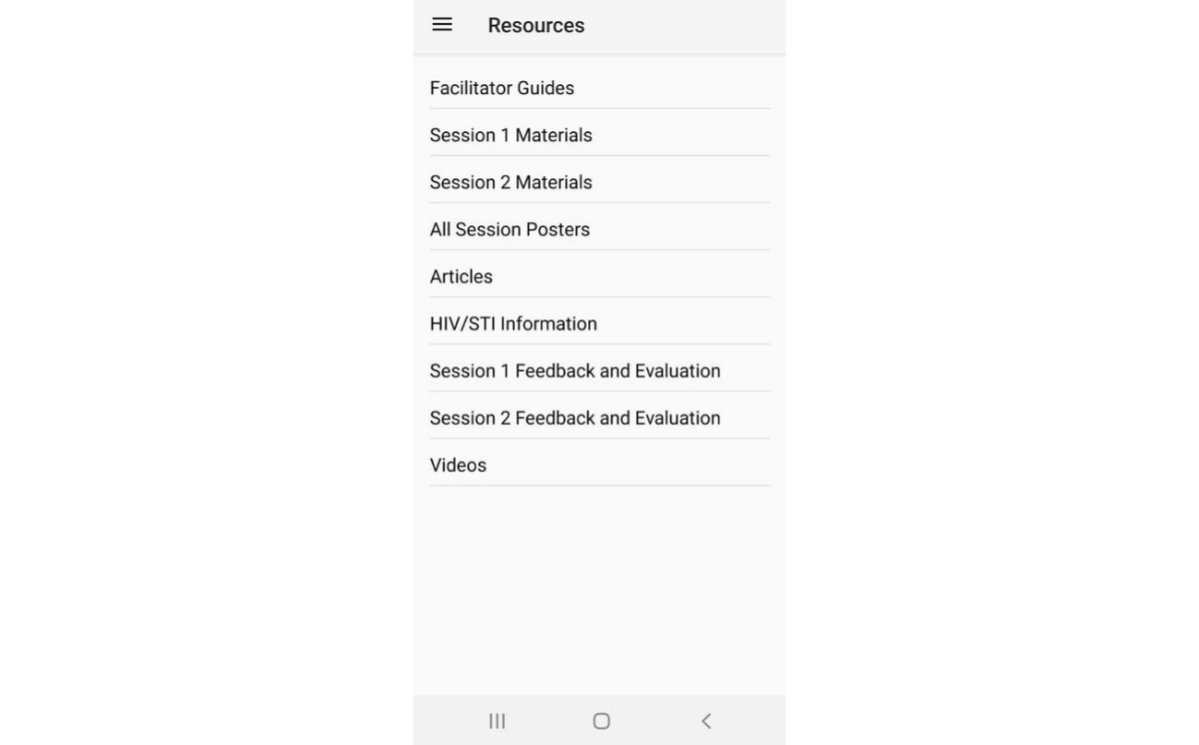
List of resource tabs in the smartphone app. STI: sexually transmitted infection.

The final prototype of the PHAT Life technology-based training tool was available for both iOS and Android operating systems. The five modules were completed sequentially, but a menu-driven option allowed participants to review previously completed material (see Figure 2). Audio narration allowed participants to listen to the written text, and each page was supplemented by a video demonstration or relevant photograph. Games and questions were interspersed throughout the training to increase user engagement (see Figure 3). At the end of the module, participants completed quizzes to evaluate knowledge learned. The training tool was designed so participants could upload video role plays facilitating curriculum activities. Expert trainers who viewed the videos could insert time-stamped comments to alert participants to segments associated with the feedback (see Figure 4). The tool provided a “Resource” tab with supplemental PHAT Life materials and links to research articles (see Figure 5), and messaging between the trainers and participants was enabled.

### Participants

Participants were 12 staff members aged 26-54 (mean 36.67, SD 9.45) years recruited from organizations that provide detention alternative services to justice-involved youth. Staff were eligible for the pilot study if they provided group-based services to youth. Table 2 provides demographic information about the sample. A total of 67% (n=8) of participants reported prior experience providing youth with sexual health information. One participant left their job and withdrew from the study, but the remainder (n=11) provided written and verbal feedback about the technology-based training platform. We deliberately enrolled 3 staff with previous in-person PHAT Life training experience to explore the perceived advantages and disadvantages of the different approaches.

**Table 2:** Sample demographics (N=12).

Characteristics		Participants, n (%)
**Gender**		
	Male	3 (25)
	Female	9 (75)
**Ethnicity**		
	Spanish/Hispanic/Latinx	4 (33)
	Not Spanish/Hispanic/Latinx	7 (58)
**Educational level**		
	High school graduate or equivalent (eg, GED^a^), some college (no degree), associate’s degree/trade school certificate, or occupational degree	3 (25)
	Bachelor’s degree (BS, BA, etc)	3 (25)
	Master’s degree or above (MA, MSW, MD, JD, etc or doctoral degree)	6 (50)
**Length of current employment**		
	1 month to 6 months	3 (25)
	6 months to 1 year	2 (16)
	1 year to 2 years	1 (8)
	>2 years	6 (50)

^a^GED: General Educational Development.

### Procedures

Participants were recruited from two sites likely to deliver PHAT Life. Eligible staff received study information via email, and interested individuals were given a link to the consent form and baseline survey. Participants were instructed to log on to the training tool and complete modules 1 to 5 (in order) at their own pace. Following modules 4 and 5, participants uploaded or conducted a live demonstration of two PHAT Life activities: anatomy and STI information. Participants could send messages to the PHAT Life trainers within the training tool. Expert trainers reviewed the role plays and quizzes, and assigned fidelity scores. All participants received live supervision where expert trainers provided feedback about the role plays and quiz responses, answered questions, and solicited comments about the intervention and training tool. Participants completed a posttraining survey evaluating HIV and STI knowledge, confidence delivering sex education, and perceptions about the tool (adoption, implementation, acceptability, appropriateness, sustainability, and satisfaction). They received a US $150 electronic gift card as compensation. All procedures were approved by the Oregon Research Institute Institutional Review Board.

### Measures

Participants completed surveys at baseline and posttraining via Qualtrics.

#### Demographics

At baseline, participants reported their age, sex/gender, educational level, experience delivering youth-related services, length of employment at the facility, and job roles and tasks.

#### Use

Computer-recorded use data tracked training attrition and dosage, namely, time at log on, length of time on the website, number of times logged on during the training period, and length of time spent on each module.

#### Participant Outcomes

At baseline and posttraining, participants completed the HIV Knowledge Questionnaire, an 18-item true/false measure of HIV prevention [46] with good internal consistency in this study (α=.75), and the STI Knowledge Survey*,* a 12-item true/false measure developed for this study that showed good internal consistency (α=.70). For both measures, total scores were derived by summing the correct answers, and this total score was used in data analyses. The Sex Education Confidence Scale assessed comfort teaching sex education on a 6-point Likert scale from 1 (“It’s not an appropriate topic”) to 6 (“Very confident, including leading discussion and answering questions”) and has strong reliability for this study (α=.94) [47].

#### Implementation Outcomes

Participants reported on implementation outcomes, including adoption/uptake, implementation, acceptability, appropriateness, sustainability, satisfaction, and fidelity [48-50]. For adoption/uptake, they indicated whether PHAT Life could be adopted in their setting and whether it met the needs of the youth they serve on a 12-item scale ranging from 1 (strongly disagree) to 5 (strongly agree). Reliability in this study was strong (α=.79), and a total score was used in the analysis. Five items assessed implementation on a scale from 1 (not at all) to 10 (very much) indicating whether PHAT Life and the technology-based training tool could be implemented in their setting. This measure had excellent reliability in this sample (α=.93).

For acceptabilit*y*, six items on a 4-point Likert scale (1=not at all to 4=a lot) measured whether the training tool was acceptable for learning the material, activities, and strategies to facilitate PHAT Life. Scale reliability was good (α=.80). Acceptability was also evaluated using computer use data related to attrition, component completion, and amount of time spent online and in supervision. To assess appropriateness, participants responded to 5 items on a scale from 1 (not at all) to 4 (a lot) to indicate whether PHAT Life is consistent with their own and their organization’s values, and addresses concerns within the juvenile justice community. The reliability was strong in this study (α=.85). For sustainability, participants responded to six items on a scale from 1 (too little or no extent) to 7 (to a very great extent) to indicate whether their organization had the infrastructure and leadership support to continue PHAT Life implementation. The scale reliability was strong (α=.88).

Participants indicated their satisfaction with the technology-based training on 21 items on a 5-point Likert scale (1=strongly disagree to 5=strongly agree). Responses were averaged to determine overall satisfaction for use in analyses. Scale reliability was strong (α=.88). To assess fidelity to the intervention, expert trainers rated participant adherence and competence based on recorded videos or live observations. For each activity, the trainer rated whether the task was completed (yes/no) and the quality of the delivery on a scale from 0 (not very well) to 4 (excellent). Trainers also rated facilitator leadership skills (eg, explained the activity correctly, was open and nonjudgmental), how smoothly the activity went, how well facilitators knew and delivered the material, and facilitator comfort on a scale from 0 (not very well) to 4 (excellent).

## Results

### Use

The pilot test occurred over 13.1 weeks. Participants spent an average of 4.3 (SD 2.2) hours to complete the training. All participants completed the five modules, two role plays, and one supervision session. Most participants preferred to use their computer to complete the training (n=7, 64%), but 27% (n=3) used a combination of the computer and smartphone app. Only 9% (n=1) used the browser on their phone exclusively. Participants spent 70% (33.17 hours) of their time using the tool during the day (8 AM to 5 PM), 20% (9.54 hours) after the workday (5 PM to 9 PM), 9% (4.61 hours) in the evening (9 PM to 12 AM), and 1% (0.43 hours) at night (12 AM to 8 AM). In total, participants exchanged 67 messages with expert trainers within the tool, primarily focused on scheduling supervision sessions.

### Participant Outcomes

**Figure 6:**
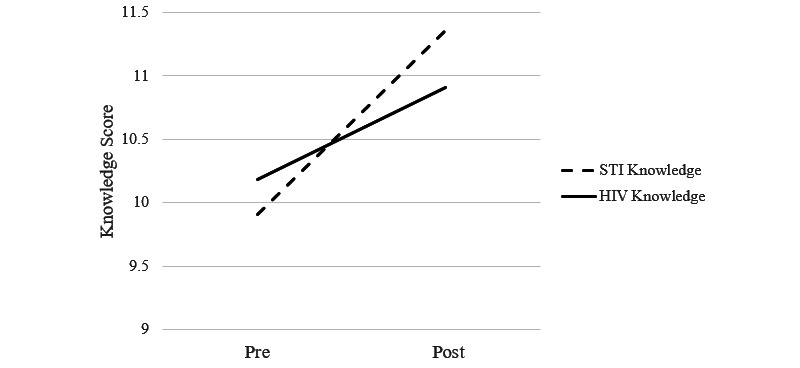
Change in STI and HIV knowledge. STI: sexually transmitted infection.

Participant STI knowledge significantly increased from baseline to posttraining (t_10_=3.07; *P*=.01; see Figure 6). Participants also showed slight improvements in HIV knowledge (t_10_=1.70; *P*=.12), although this did not reach statistical significance. The difference in the paired *t* test for HIV knowledge resulted in a Cohen *d* of 0.63, which is considered a moderate to large effect size. Quizzes at the end of each module indicated that most participants learned the material. A quiz score of 80% was considered passing, and 80% (n=9) of participants passed modules 1, 3, and 4; all participants passed module 2; and 90% (n=10) passed module 5. At baseline, participants reported high confidence in providing sexual education to youth (mean 5.14, SD 0.67), but this diminished following the training (mean 4.74, SD 0.68; t_10_=2.51; *P*=.03).

### Implementation Outcomes

On average, participants *strongly agreed* that PHAT Life could be adopted (mean 4.26, SD 0.42) and implemented (mean 9.07, SD 0.87) in their setting to meet the needs of the youth served. They rated the training as very acceptable (mean 3.74, SD 0.32) and appropriate (mean 3.80, SD 0.32), and highly sustainable in their settings (mean 6.02, SD 0.94). They described the videos (mean 4.82, SD 0.41), manuals (mean 4.73, SD 0.47), role play activities (mean 4.64, SD 0.67), and supervision (mean 4.73, SD 0.47) as very useful. Few technical issues were reported, and there was general agreement that the tool was easy to use and navigate. Some participants expressed difficulty uploading videos of the role plays and preferred live demonstrations. Of the 3 participants who previously received the in-person PHAT Life training, 2 rated the in-person and technology-based training equally and 1 preferred the in-person training to be able to ask questions in the moment. The mean rating for satisfaction posttraining (mean 4.42, SD 0.36) indicated high satisfaction, and this was supported by participants’ open-ended comments:

The online platform was excellent, well-planned, and well-developed.

I liked how everything was explained in writing and in a video message.

I liked the step-by-step process of describing the activity and then having a video example of the activity in action.

It gives good information on obtaining and retaining youth attention and keeping them engaged.

Fidelity to the curriculum was excellent (mean 3.39, SD 0.62) with high ratings for adherence (mean 3.71, SD 0.78) and competence (mean 3.38, SD 0.87). Supervision sessions lasted 20 to 60 minutes. Participants were asked on the postsurvey how often they preferred individual supervision to inform the training model; 55% (n=6) said once a year or every 6 months, and 36% (n=4) endorsed more frequent supervision. Only 1 participant said they did not need ongoing supervision.

## Discussion

### Principal Results

This study describes the development and pilot testing of a prototype technology-based training tool for PHAT Life, a comprehensive health promotion and sex education EBP for justice-involved youth [21]. Creation of the training tool required strong collaboration between research and industry teams, and findings support the use of online training as a strategy to address a critical implementation barrier to the uptake of EBPs for justice-involved youth. Results demonstrated feasibility and warrant completion of the PHAT Life technology-based training tool.

Consistent with the hypothesis, participant knowledge of HIV and STI increased as reflected in their high scores on module quizzes and pre/posttraining measures. In addition, participants performed the two session activities with high fidelity. Surprisingly, HIV knowledge did not significantly increase from pre- to posttraining, but a trend emerged. Three possible reasons may explain the lack of significant increase in HIV knowledge. First, it is possible that role playing teaching the STI activity for evaluation by the expert trainer facilitated learning about STIs more so than HIV. Indeed, evidence supports that teaching improves knowledge [51]. Second, it is also possible that the two sessions in the prototype lacked sufficient information to significantly improve HIV knowledge among participants. Lastly, since participants were highly knowledgeable about HIV at pretest (ie, they answered 10 of 12 questions correctly), there was little room for improvement. Overall, results of this pilot test suggest that technology-based training may be sufficient to learn new information and achieve intervention fidelity.

Contrary to expectations, sex education confidence significantly decreased from pre- to posttraining. It is possible that participants overestimated their knowledge about sexual health at baseline and once exposed to the training material became aware of their knowledge deficits. For example, one participant noted, “I didn’t know this before,” when reflecting on topics related to male and female anatomy and STIs. The reduced confidence may be temporary, however; once participants deliver the intervention, their confidence and self-efficacy will likely improve [52].

Consistent with expectations, participants rated the technology-based training tool positively across all implementation outcomes. This is highly encouraging because in-person training can be a significant barrier to uptake, adoption, and dissemination of EBPs [53,54]. Moreover, other advantages of a technology-based training tool exist that could diminish implementation barriers raised by CFIR [32]. First, it eliminates variability across trainings since the tool is the same for all learners (ie, intervention characteristics) [32]. Second, a technology-based tool can be accessible at any time of the day (or night) to accommodate the learner and the setting’s scheduling needs (ie, characteristics of individuals and inner setting) [32]. Third, it does not require group-based meetings that could interfere with organizational operations by pulling staff from their typical job responsibilities (ie, inner setting). Finally, a technology-based tool can be accessed on different platforms (phone app, computer) from which learners can choose depending on what is most comfortable and accessible (ie, characteristics of individuals). A technology-based tool can reduce inner setting barriers by limiting interference with daily work responsibilities and by providing opportunities to quickly train new staff.

### Limitations

It is important to note that the results of this study are preliminary, and the sample size was small. Once the tool is fully built, it will be important to evaluate the same participant and implementation outcomes with a larger sample and to diversify the learner pool to include potential facilitators not included here. Fidelity was achieved in this pilot study, but only for two curricular activities. The next step will be to examine fidelity to the full training. Most participants used a computer to complete the training, and this limited feedback about the tool’s utility using the phone app. Increasing app use will offer much-needed information to refine this training approach. Two-thirds of participants reported prior experience providing sexual health information to youth. The broad nature of the question (ie, have you had experience providing sexual health information to youth?) limits our understanding of the kind and type of prior experience. It is possible that inexperienced participants would respond differently to the training. As a next step, once the platform is fully developed, increasing the sample size, implementing a rigorous randomization schedule, and widening the sample to include individuals without prior experience in sexual health will inform generalizability.

### Future Directions

Several lessons from the pilot study will guide adaptations as we complete the training tool. Participants request embedded links to connect them with additional information about related topics (eg, sexual trauma) and skill development. In the final tool, we will include supplementary resources and links to key topics. We observed that some participants skipped the videos, and these omissions appeared to diminish knowledge acquisition. We will require all participants to watch the videos before moving on to the next module. Some learners reported missing peer-to-peer interactions during training. For the final tool, we will create a forum for peer-to-peer support and communication.

The COVID-19 pandemic has normalized the use of technology for many activities, including health care delivery, workplace meetings, educational conferences, and university teaching. This pilot study provides strong preliminary evidence that a technology-based tool can effectively train individuals to deliver an EBP with fidelity, and that this implementation strategy is satisfying, appropriate, and sustainable with justice-involved settings. If replicated with a larger sample, this approach addresses a key barrier to adoption and implementation of EBPs.

## Abbreviations

CFIR: Consolidated Framework for Implementation Research
EBP: evidence-based program
NIMHD: National Institute of Minority Health and Health Disparities
PHAT: Preventing HIV/AIDS Among Teens
STI: sexually transmitted infection
